# Outstanding Electrochemical Performance of Ni-Rich Concentration-Gradient Cathode Material LiNi_0.9_Co_0.083_Mn_0.017_O_2_ for Lithium-Ion Batteries

**DOI:** 10.3390/molecules28083347

**Published:** 2023-04-10

**Authors:** Hechen Li, Yiwen Guo, Yuanhua Chen, Nengshuang Gao, Ruicong Sun, Yachun Lu, Quanqi Chen

**Affiliations:** 1 Guangxi Key Laboratory of Electrochemical and Magneto-Chemical Functional Materials, College of Chemistry and Bioengineering, Guilin University of Technology, Guilin 541004, China; 2120200819@glut.edu.cn (H.L.);; 2 College of Mechanical and Vehicle Engineering, Hunan University, Changsha 410082, China; 3 School of Automobile Engineering, Guilin University of Aerospace Technology, Guilin 541004, China

**Keywords:** concentration-gradient, Ni-rich ternary, cathode materials, lithium-ion batteries

## Abstract

The full-concentrationgradient LiNi_0.9_Co_0.083_Mn_0.017_O_2_ (CG-LNCM), consisting of core Ni-rich LiNi_0.93_Co_0.07_O_2_, transition zone LiNi_1−x−y_Co_x_Mn_y_O_2,_ and outmost shell LiNi_1/3_Co_1/3_Mn_1/3_O_2_ was prepared by a facile co-precipitation method and high-temperature calcination. CG-LNCM was then investigated with an X-ray diffractometer, ascanning electron microscope, a transmission electron microscope, and electrochemical measurements. The results demonstrate that CG-LNCM has a lower cation mixing of Li^+^ and Ni^2+^ and larger Li^+^ diffusion coefficients than concentration-constant LiNi_0.9_Co_0.083_Mn_0.017_O_2_ (CC-LNCM). CG-LNCM presents a higher capacity and a better rate of capability and cyclability than CC-LNCM. CG-LNCM and CC-LNCM show initial discharge capacities of 221.2 and 212.5 mAh g^−1^ at 0.2C (40 mA g^−1^) with corresponding residual discharge capacities of 177.3 and 156.1 mAh g^−1^ after 80 cycles, respectively. Even at high current rates of 2C and 5C, CG-LNCM exhibits high discharge capacities of 165.1 and 149.1 mAh g^−1^ after 100 cycles, respectively, while the residual discharge capacities of CC-LNCM are as low as 148.8 and 117.9 mAh g^−1^ at 2C and 5C after 100 cycles, respectively. The significantly improved electrochemical performance of CG-LNCM is attributed to its concentration-gradient microstructure and the composition distribution of concentration-gradient LiNi_0.9_Co_0.083_Mn_0.017_O_2_. The special concentration-gradient design and the facile synthesis are favorable for massive manufacturing of high-performance Ni-rich ternary cathode materials for lithium-ion batteries.

## 1. Introduction

Compared with other secondary batteries such as lead acid, nickel–cadmium and nickel–metal hydride batteries, lithium-ion batteries (LIBs) have been widely used in portable electronic devices and electric vehicles (EVs) because of their higher energy density and longer life span [1,2,3,4,5,6]. With the increasing demand for LIBs with a high energy density and outstanding cyclability, it is urgent and imperative to develop high-energy density and cyclability of cathode materials for LIBs. Numerous efforts have been made to optimize cathode materials, and dozens of these have been well developed [7], but the Ni-based ternary cathode materials LiNi_1−x−y_Co_x_Mn_y_O_2_ (NCM) and LiNi_1−x−y_Co_x_Al_y_O_2_ (NCA) have received extensive and intensive attention and have been extensively utilized [8,9,10,11,12,13]. Ni-rich ternary cathode materials supply a large capacity that rises proportionately with Ni content, even though they suffer from inferior cyclic stability during cycling and thermal instability [14,15,16,17,18]. These shortcomings mainly result from the microcracking of electroactive particles upon the charging/discharging processes, which tremendously enlarges the inner surface exposed to electrolyte attack [15,19].

To improve the performance of Ni-rich ternary cathode materials, diverse approaches such as doping heterogeneous ions [20,21,22], surface coating [23,24,25], the construction of a single crystal phase [26,27,28], core/shell microstructure [29,30] and particles with concentration-gradient composition [31,32,33] have been adopted to inhibit the formation of microcracking. Among the preceding approaches, building particles with concentration-gradient composition is one of the most effective ways to improve the cyclability of electroactive materials. Generally, core composition can provide high capacity, and shell composition can improve cyclability and thermal stability in the ideal concentration-gradient microstructure [34]. In the present investigation, full concentration-gradient LiNi_0.9_Co_0.087_Mn_0.013_O_2_, in which the composition varies gradiently from core composition LiNi_0.93_Co_0.07_O_2_ to outmost shell composition LiNi_1/3_Co_1/3_Mn_1/3_O_2_, was proposed and prepared by a facile co-precipitation method combined with high-temperature calcination in an oxygen atmosphere. Benefiting from the high capacity provided by Ni-rich core composition and the excellent cyclability of shell composition LiNi_1/3_Co_1/3_Mn_1/3_O_2_, the concentration-gradient LiNi_0.9_Co_0.087_Mn_0.013_O_2_ exhibits significantly improved electrochemical performance in comparison with the concentration-constant LiNi_0.9_Co_0.087_Mn_0.013_O_2_ prepared by the same synthesis procedure.

## 2. Results and Discussions

**Figure 1:**
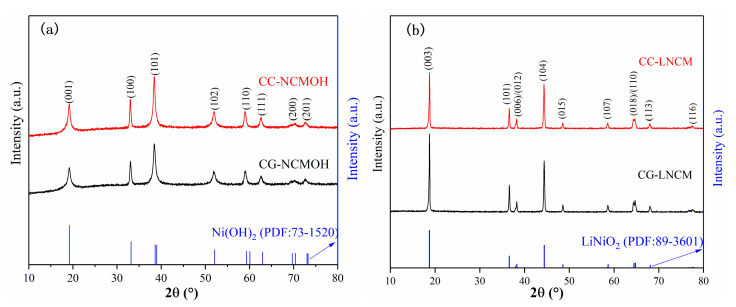
XRD patterns of (**a**) CG-NCMOH and CC-NCMOH; (**b**) CG-LNCM and CC-LNCM.

Figure 1 shows the X-ray diffraction (XRD) patterns of concentration-gradient precursors (CG-NCMOH), concentration-constant precursors (CC-NCMOH), the concentration-gradient products (CG-LNCM), and the concentration-constant products (CC-LNCM). Figure 1a and the Rietveld refinement results (Figure S1a,b in Supplementary File) demonstrate that no manganese and cobalt hydroxides or other oxides exist in the XRD patterns of hydroxide precursors CC-NCMOH and CG-NCMOH, indicating that Mn^2+^ and Co^2+^ are successfully doped into Ni sites of Ni(OH)_2_ to form Ni-Co-Mn ternary hydroxide. The diffraction peaks of CC-NCMOH and CG-NCMOH shift to a lower angle, which implies that doping of Mn^2+^ and Co^2+^ enlarges the layer distance of Ni(OH)_2_ according to the Bragg equation 2dsin θ = nλ. As observed in Figure 1b, the diffraction peaks of CC-LNCM and CG-LNCM are strong and sharp, suggesting that both CC-LNCM and CG-LNCM have high crystallinity, and all the diffractions can be well indexed by the R3m space group. The structural parameters for CC-LNCM and CG-LNCM are listed in Tables S1 and S2, and the results reveal that CG-LNCM has a larger cell volume than CC-LNCM, hinting that CG-LNCM may have better electrochemical performance in comparison with CC-LNCM because a larger cell volume favors the more rapid transport of Li^+^ in electroactive particles and hence the better electrochemical performance. The layered structure of the material could be judged by the splitting of characteristic peaks. The more obvious the splits of (006)/(102) and (018)/(110) are, the more they indicate a higher degree of layered structure [35]. In addition, the ratio of c/a for both CC-LNCM and CG-LNCM is bigger than 4.9, showing that both samples have a well-developed layered crystal structure [36,37]. Furthermore, the quota of intensity ratio of (003) to (104), I_(003)_/I_(104)_ reflects the cation mixing of Li^+^ and Ni^2+^, and the larger value of I_(003)_/I_(104)_ means the lower cation mixing. It was reported that if the value of I_(003)_/I_(104)_ is bigger than 1.2, the materials will have lower cation mixing [38,39] and improved electrochemical performance. According to Tables S1 and S2, the comparisons of CC-LNCM and CG-LNCM in I_(003)_/I_(104)_ and the occupation of Li and Ni in Wyckoff sites 3a and 3b reveal that CG-LNCM has a lower cation mixing of Li^+^ and Ni^2+^ than CC-LNCM, and the special concentration-gradient microstructure may be responsible for the lower cation mixing.

**Figure 2:**
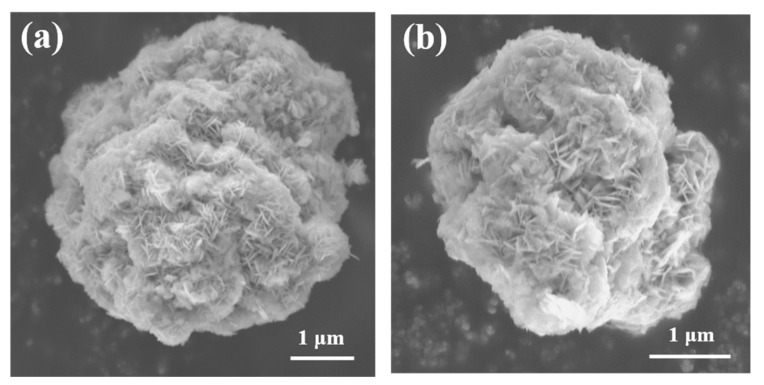

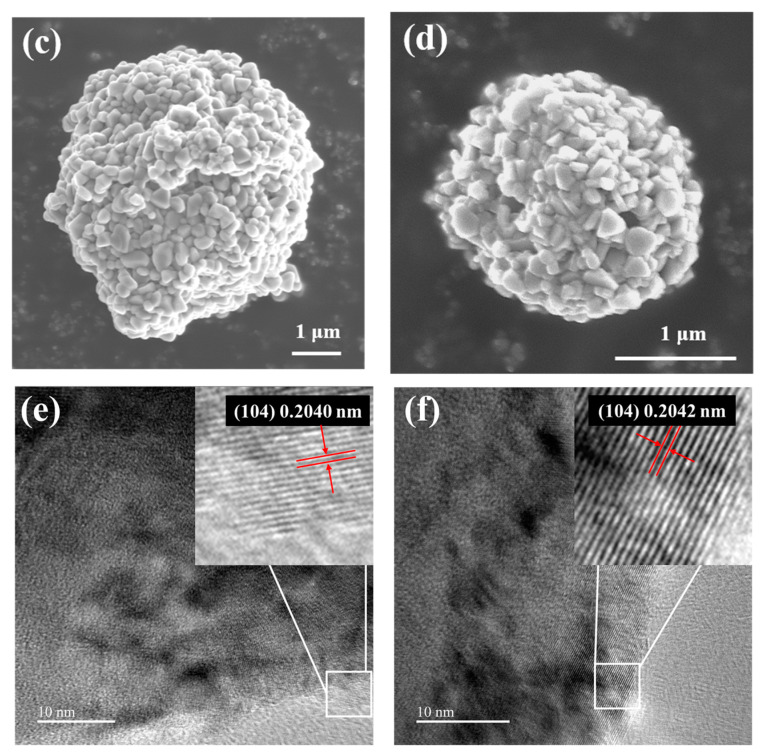
SEM images of (**a**) CC-NCMOH, (**b**) CG-NCMOH, (**c**) CC-LNCMO and (**d**) CG-LNCM; HRTEM images of (**e**) CC-NCMOH and (**f**) CG-NCMOH.

The scanning electron microscope (SEM) images of CC-NCMOH, CG-NCMOH, CC-LNCM and CG-LNCM are presented in Figure 2. As observed in Figure 2a,b, both two hydroxide precursors display sphere-like morphology and a rough surface and are composed of nanoplates agglomerated loosely together. The loose agglomeration of CC-NCMOH and CG-NCMOH is beneficial for rapid reaction with LiOH to form LiNi_0.9_Co_0.083_Mn_0.017_O_2_ with high crystallinity. Figure 2c,d demonstrate that the final products CC-LNCM and CG-LNCM present a similar sphere-like morphology to that of hydroxide precursors. However, unlike the precursors CC-NCMOH and CG-NCMOH, CC-LNCM and CG-LNCM consist of nanoparticles. Compared with the compact CC-LNCM, the CG-LNCM particles are loosely agglomerated, which is favorable for the permeation of the electrolyte and rapid diffusion of Li^+^ in the electroactive particles. The high-resolution transmission electron microscope (HRTEM) images of CC-LNCM and CG-LNCM are shown in Figure 2e,f, respectively, and d-spacing of about 0.2040 and 0.2042 nm is observed in the lattice fringes of CC-LNCM and CG-LNCM, respectively, corresponding to the lattice plane (104). The minor difference of the d-space may result from the different surface composition of LiNi_0.9_Co_0.083_Mn_0.017_O_2_ and LiNi_1/3_Co_1/3_Mn_1/3_O_2_ for CC-LNCM and CG-LNCM, respectively.

**Figure 3:**
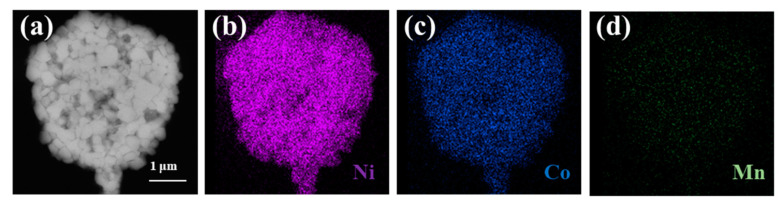

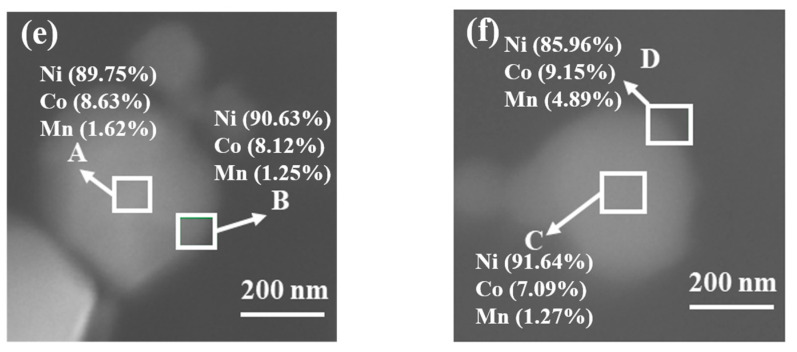
SEM image of cross-section of (**a**) CG-LNCM and the corresponding EDX elemental mappings of (**b**) Ni, (**c**) Co and (**d**) Mn; SEM images of primary particles and compositions of the selected area of (**e**) CC-LNCM and (**f**) CG-LNCM.

The SEM image of cross-section of CG-LNCM presented in Figure 3a further clearly demonstrates that the sphere-like secondary particles are composed of primary nanoparticles, and the corresponding EDX elemental mappings in Figure 3b–d indicate that the elements Ni, Co and Mn are evenly dispersive, similar to the concentration-constant LiNi_0.8_Co_0.1_Mn_0.1_O_2_ [36], implying that it is difficult to separate concentration-gradient samples from concentration-constant samples by elemental mappings. Figure 3e,f display the SEM images of CC-LNCM and CG-LNCM primary particles, respectively. The element contents of the selected area of particles are determined by energy dispersive X-ray spectroscopy (EDXS), and EDXS mappings of the selected area are shown in Figure S2. It can be observed that contents of Ni, Co, and Mn in the edge and interior of the primary particle are almost identical and close to the molar ratio of Ni, Co and Mn of the concentration-constant LiNi_0.9_Co_0.083_Mn_0.017_O_2_. The compositions are different in the different areas of the CG-LNCM, further confirming that composition varies gradiently from interior to edge and CG-LNCM is truly a concentration-gradient oxide.

**Figure 4:**
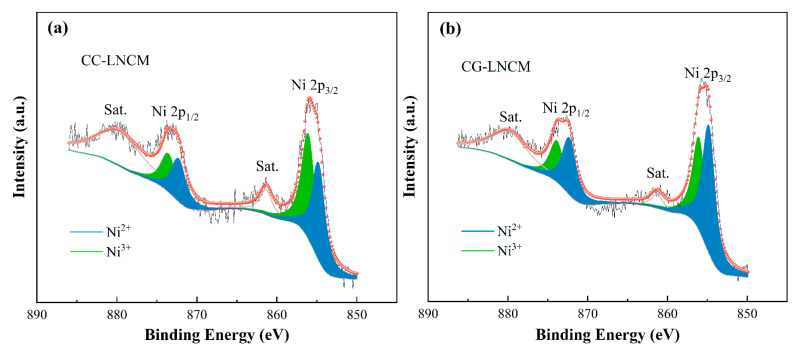
Ni 2p XPS spectra of (**a**) CC-LNCM and (**b**) CG-LNCM.

To learn the effects of the concentration-gradient and concentration-constant microstructure on the oxidate state of Ni in LiNi_0.9_Co_0.083_Mn_0.017_O_2_, an X-ray photoelectron spectroscopy (XPS) measurement was conducted and the corresponding Ni 2p XPS spectra were shown in Figure 4. As can be observed in Figure 4a,b, the XPS spectra of both CC-LNCM and CG-LNCM consist of two satellite peaks and two main peaks, and the two peaks centered at about 855.96 and 873.5 eV are deconvoluted into 856.1 and 854.9 eV, and 873.9 and 872.4 eV, respectively. The Binding energy of 854.9 and 872.4 eV correspond to Ni 2p_3/2_ and Ni 2p_1/2_ of Ni(II) [40,41], respectively, and the binding energy of 856.1 and 873.9 eV match Ni 2p_3/2_ and Ni 2p_1/2_ of Ni(III) [40,42], respectively. The molar ratio of Ni^2+^/Ni^3+^ is calculated according to the ratio of the closed area of Ni 2p_3/2_ XPS spectra of Ni^2+^ to the closed area of Ni 2p_3/2_ XPS spectra of Ni^3+^. The molar ratios of Ni^2+^/Ni^3+^ are 0.48/0.52 and 0.58/0.42 for CC-LNCM and CG-LNCM, respectively. The different content of Ni^2+^ results from the different surface compositions of CC-LNCM and CG-LNCM, similar to the previous report that the molar ratio of Ni^2+^/Ni^3+^ is larger on the surface of LiNi_1−x−y_Co_x_Mn_y_O_2_ with a lower content of Ni [43]. This finding further confirms the concentration-gradient microstructure of CG-LNCM, in which the composition varies from the core LiNi_0.93_Co_0.07_O_2_ to the outmost shell LiNi_1/3_Co_1/3_Mn_1/3_O_2_.

**Figure 5:**
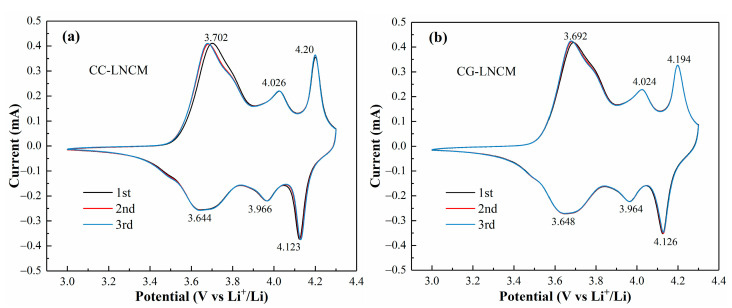
The first three cycles of cyclic voltammograms of (**a**) CC-LNCM and (**b**) CG-LNCM at 0.1 mV s^−1^ in the potential range of 3.0–4.3 V (vs. Li^+^/Li).

To investigate the effect of composition distribution of LiNi_0.9_Co_0.083_Mn_0.017_O_2_ on the electrochemical mechanism, cyclic voltammetry measurements were conducted on CC-LNCM and CG-LNCM at a scan rate of 0.1 mV s^−1^ in the potential scope of 3.0–4.3 V (vs. Li^+^/Li) at room temperature, and the first three cycles of cyclic voltammograms (CVs) of CC-LNCM and CG-LNCM electrodes are shown in Figure 5. The shape and the closed area of the first three CVs are almost unchanged, suggesting that both CC-LNCM and CG-LNCM have the better cyclability. As seen in Figure 5a,b, two CVs exhibit a similar shape, and three couples of redox peaks have close potential, indicating that CC-LNCM and CG-LNCM possess the identical electrochemical mechanism upon the charging and discharging processes. The three redox peaks are associated with the interconversion of the hexagonal phase to the monoclinic phase (H1↔M), the monoclinic phase to the hexagonal phase (M↔H2), and the hexagonal phase to the hexagonal phase (H2↔H3), respectively [44,45]. The accurate comparison of redox peak potentials of CC-LNCM and CG-LNCM demonstrates that CG-LNCM has lower oxidation peak potentials and larger reduction peak potentials than CC-LNCM, implying that CG-LNCM exhibits lower electrochemical polarization and better electrochemical reaction reversibility than CC-LNCM.

**Figure 6:**
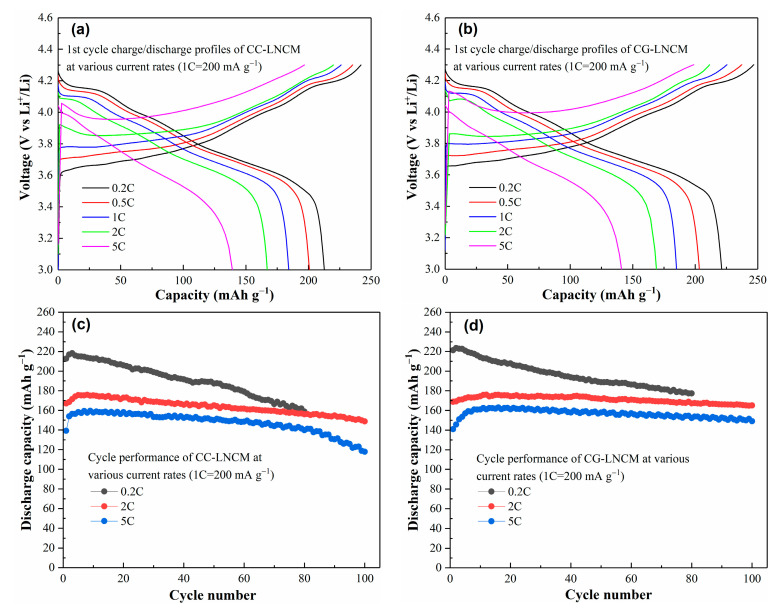
Initial charge/discharge profiles of (**a**) CC-LNCM and (**b**) CG-LNCM at various current rates, cycle performance of (**c**) CC-LNCM and (**d**) CG-LNCM at various current rates.

The electrodes used to evaluate galvanostatic electrochemical performance at each given current rate were fresh CC-LNCM or CG-LNCM electrodes without an activation process. The initial charge/discharge profiles of CC-LNCM and CG-LNCM electrodes at various current rates (1C = 200 mA g^−1^) in the voltage range of 3.0–4.3 V (vs. Li^+^/Li) were shown in Figure 6a,b, and the big difference indicated between charge and discharge capacities is observed. The low initial Coulombic efficiency of fresh CC-LNCM and CG-LNCM electrodes, similar to that of LiNi_0.9_Co_0.08_Al_0.02_O_2_ [45], may be associated with properties of Ni-rich based oxide cathode materials. Low initial Coulombic efficiency is presented in rich Ni-based oxide cathode materials, especially nanoparticles. After several cycles, the Coulombic efficiency greatly increases, as observed in Figure S3, to about 97.8% in the fifth cycle, implying that the activation of electrodes can remarkably improve the Coulombic efficiency. It can be found that the discharge and charge capacities decrease with the increase of current rates due to a larger polarization at higher current rates. The initial discharge capacities of CC-LNCM are 212.5, 200.7, 183.9, 167.1 and 139.2 mAh g^−1^ at 0.2C, 0.5C, 1C, 2C and 5C, respectively, while CG-LNCM presents the somewhat higher initial discharge capacities of 221.2, 203.3, 185.0, 168.9 and 140.8 mAh g^−1^ at 0.2C, 0.5C, 1C, 2C and 5C, respectively. The initial discharge capacity of CC-LNCM at 0.2C is close to that of 210 mAh g^−1^ for LiNi_0.9_Co_0.05_Mn_0.05_O_2_ at 0.2C [37], higher than that of 203.8 mAh g^−1^ for LiNi_0.91_Co_0.06_Mn_0.03_O_2_ single crystal at 0.1C [28], 207 mAh g^−1^ for LiNi_0.9_Co_0.08_Al_0.02_O_2_ at 0.2C [46]. The initial discharge capacity of CG-LNCM at 0.2C is close to that of about 221 mAh g^−1^ for concentration-gradient LiNi_0.84_Co_0.06_Mn_0.09_Al_0.01_O_2_ at 0.1C [32], and larger than that of 200 mAh g^−1^ for concentration-gradient LiNi_0.9_Mn_0.1_O_2_ at 0.1C [47]. The initial discharge capacities of CG-LNCM at 0.5C, 1C and 2C are close to those of 200 mAh g^−1^ for LiNi_0.92_Co_0.03_Mn_0.03_Al_0.02_O_2_ at 0.5C [48], 180 mAh g^−1^ for TiO_2_-coated LiNi_0.9_Co_0.08_Al_0.02_O_2_ at 1C [47] and 165 mAh g^−1^ for TiO_2_-coated LiNi_0.9_Co_0.08_Al_0.02_O_2_ at 2C [45], respectively. The above-mentioned results demonstrate that CG-LNCM is competitive in the first discharge capacity with other Ni-based ternary cathode materials.

The cycle performance of CC-LNCM and CG-LNCM at 0.2C, 2C and 5C are depicted in Figure 6c,d. As observed in Figure 6c,d, discharge capacities of CC-LNCM and CG-LNCM at all current rates increase before the first number of specific cycles, which is ascribed to the activation process that originates from the insufficient contact between electrolyte and electroactive particles. After activation, the discharge capacity of CC-LNCM reaches the highest value of 218.3 mAh g^−1^ at 0.2C and decreases to 156.1 mAh g^−1^ after 80 cycles. The ratio of the residual discharge capacity to the highest discharge capacity corresponds to 71.4%. While CG-LNCM exhibits the highest capacity of 223.4 mAh g^−1^ in the third cycle and higher residual capacity of 177.3 mAh g^−1^ after 80 cycles at 0.2C, the ratio of the residual discharge capacity to the highest discharge capacity corresponds to 79.4%. The residual capacity of CG-LNCM at 0.2C is close to that of about 174 mAh g^−1^ for LiNi_0.9_Co_0.08_Al_0.02_O_2_ [46], about 170 mAh g^−1^ for TiO_2_-coated LiNi_0.9_Co_0.08_Al_0.02_O_2_ [45], and 169 mAh g^−1^ for LiTaO_3_ modified LiNi_0.9_Co_0.06_Mn_0.04_O_2_ [49] after 80 cycles at 0.2C. The residual discharge capacities of CC-LNCM are 148.8 and 117.9 mAh g^−1^ at 2C and 5C after 100 cycles, respectively, which are much smaller than the corresponding residual discharge capacities of CG-LNCM. The special concentration-gradient microstructure of CG-LNCM may be responsible for the improved electrochemical performance. The residual discharge capacity of CG-LNCM at 2C is 165.1 mAh g^−1^ after 100 cycles, which is larger than that of 130 mAh g^−1^ for TiO_2_-coated LiNi_0.9_Co_0.08_Al_0.02_O_2_ [45], 134.4 mAh g^−1^ for LiNi_0.9_Co_0.05_Mn_0.05_O_2_ [50], and 156.9 mAh g^−1^ for Li_2_SiO_3_ coated LiNi_0.9_Co_0.05_Mn_0.05_O_2_ [50] at 2C after 100 cycles. Even at the high current rate of 5C, CG-LNCM presents the high discharge capacity of 149.1 mAh g^−1^ after 100 cycles, significantly higher than that of 117.9 mAh g^−1^ for CC-LNCM. It is noted that both CC-LNCM and CG-LNCM show better cycle performance at higher current rates than the cycle performance of these two cathodes at 0.2C, and this phenomenon is similar to that of LiNi_0.8_Co_0.1_Mn_0.1_O_2_ [51]. This phenomenon may be ascribed to the lower tolerance of great deformation of CC-LNCM and CG-LNCM with loose microstructure and nanosized primary particles. Compared with cycles at higher current rates, extraction of more Li^+^ from electroactive material LiNi_0.9_Co_0.083_Mn_0.017_O_2_ and insertion of more Li^+^ into delithiated LiNi_0.9_Co_0.083_Mn_0.017_O_2_ occur at lower current rates during charging and discharging processes, resulting in huge deformation, structure instability and the resultant inferior cyclability at lower current rates. In summary, the aforementioned electrochemical tests demonstrate that CG-LNCM has a higher capacity and better rate capability and cyclability than CC-LNCM, which can be attributed to the advantages of the concentration-gradient microstructure that the Ni-rich core LiNi_0.93_Co_0.07_O_2_ provides. Furthermore, the high capacity and shell composition of LiNi_1/3_Co_1/3_Mn_1/3_O_2_ supplies excellent structural stability, and the multiple core/shell structure of concentration-gradient composition is favorable for alleviation of microcracks upon cycling.

**Figure 7:**
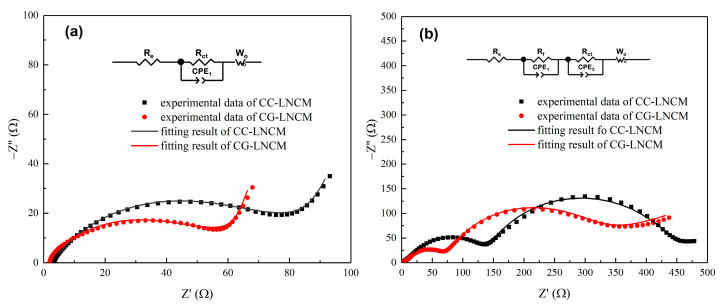
Nyquist plots of (**a**) fresh electrodes and (**b**) electrodes after cycling for 100 cycles at 5C.

To further understand the difference in electrochemical properties of concentration-gradient and concentration-constant LiNi_0.9_Co_0.083_Mn_0.017_O_2_, electrochemical impedance spectroscopy (EIS) was carried out on the fresh CG-LNCM and CC-LNCM electrodes and other electrodes that had been cycled for 100 cycles at 5 C, and the corresponding Nyquist plots are shown in Figure 7. As observed in Figure 7a, the Nyquist plots of fresh electrodes are composed of a pressed semicircle and a sloped line, which is different from the Nyquist plots (Figure 7b) of cycled electrodes that consist of two compressed semicircles and an inclined line. The equivalent circuit models for the different Nyquist plots are depicted in the insert of Figure 7a,b, respectively. In equivalent circuit models, R_e_, R_f_ and R_ct_ represent internal resistance of cell, charge transfer resistance, and resistance of the solid electrolyte interface (SEI) [52,53], respectively, while CPE and W_o_ stand for double layer capacitance and capacity of the surface layer, and Warburg impedance, respectively. The fitting results are listed in Table S3 and indicate that values of R_f_ and R_ct_ of CC-LNCM electrodes are larger than those of CG-LNCM electrodes at the corresponding states. The smaller R_ct_ of CG-LNCM electrodes suggests that CG-LNCM possesses better electrochemical kinetics and hence better electrochemical performance in comparison with CC-LNCM. In addition, the smaller R_f_ of cycled CG-LNCM electrodes demonstrates that the resistance of SEI of a cycled CG-LNCM electrode is smaller than that of a cycled CG-LNCM electrode, implying that CG-LNCM has a lower polarization than CC-LNCM during cycling, favorable for improvement of cyclability.

It is well-known that the diffusion of Li^+^ in electroactive particles is a rate-determining step of the electrochemical reaction of electroactive materials, and the Li^+^ diffusion coefficient, *D_Li_*, of electrode materials is a key parameter to evaluate the kinetics of electrochemical reaction. *D_Li_* was calculated according to the following Equation (1) [54].

(1) $$D_{Li}=\frac{4}{\pi \tau }{\left(\frac{m_{B}V_{m}}{M_{B}S}\right)}^{2}{\left(\frac{\Delta E_{s}}{\Delta E_{\tau }}\right)}^{2}$$

**Figure 8:**
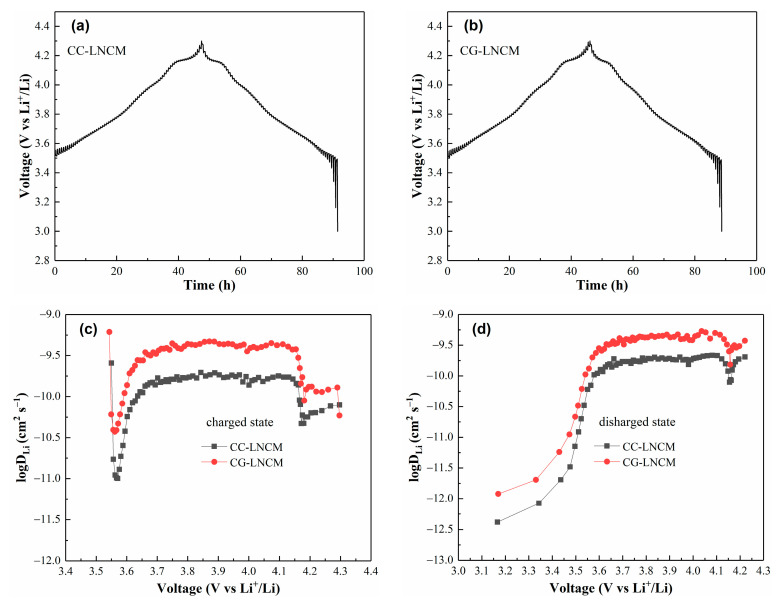
GITT curves of (**a**) CC-LNCM and (**b**) CG-LNCM, relationship between voltage and Li^+^ diffusion coefficient of CC-LNCM and CG-LNCM at (**c**) charged state and (**d**) discharged state.

The meanings of symbols in Equation (1) are the same as in our previous report, and the detailed calculation procedure is also akin to our previous report [55]. To compare *D_Li_* of CC-LNCM and CG-LNCM, the galvanostatic intermittent titration technique (GITT) measurement was carried out and the value of *D_Li_* was estimated by Equation (1). The GITT curves of CC-LNCM and CG-LNCM are shown in Figure 8a,b. The calculated values of *D_Li_* of CC-LNCM and CG-LNCM at charged and discharged states are depicted in Figure 8c,d, demonstrating that CG-LNCM has higher values of *D_Li_* in both charged and discharged states than CC-LNCM. The differences of *D_Li_* result from the discrepancies of CG-LNCM and CC-LNCM. In the charging process, the values of *D_Li_* of CG-LNCM vary from 3.73 × 10^−11^ to 6.14 × 10^−10^ cm^2^ s^−1^, while *D_Li_* of CC-LNCM lies in the scope of 1.00 × 10^−11^ to 1.96 × 10^−10^ cm^2^ s^−1^. During discharging, the CG-LNCM and CC-LNCM range in *D_Li_* from 1.20 × 10^−12^ to 5.34 × 10^−10^ and 4.15 × 10^−13^ to 2.16 × 10^−10^ cm^2^ s^−1^, respectively. The comparison of *D_Li_* in CG-NCM and CC-NCM suggests that the electrochemical reaction rate of CG-LNCM is faster than that of CC-LNCM, which can explain why CG-LNCM has a better electrochemical performance than CC-LNCM. The larger diffusion coefficients of Li^+^ benefit from the concentration-gradient composition and microstructure, and result in the significantly improved electrochemical performance of LiNi_0.9_Co_0.083_Mn_0.017_O_2_.

## 3. Materials and Methods

### 3.1. Synthesis of Materials

**Figure 9:**
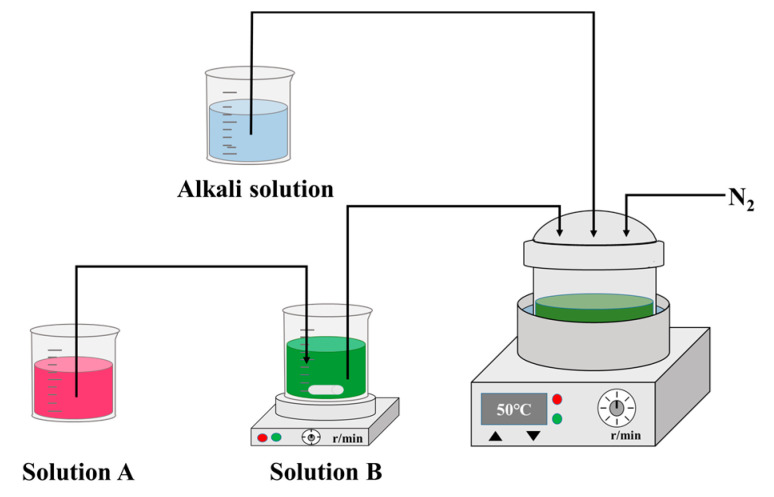
Schematic illustration of the preparation of concentration-gradient precursor Ni_0.9_Co_0.083_Mn_0.017_(OH)_2_.

The concentration-gradient precursor Ni_0.9_Co_0.083_Mn_0.017_(OH)_2_ was synthesized by a facile co-precipitation method using NiSO_4_·6H_2_O, CoSO_4_·7H_2_O, MnSO_4_·H_2_O, NaOH and NH_3_·H_2_O as raw materials, and the corresponding schematic illustration is depicted in Figure 9. To avoid the oxidation of Ni^2+^, Co^2+^ and Mn^2+^ during the synthesis of precursor Ni_x_Co_y_Mn_1−x−y_(OH)_2_ precipitate, both solution and reaction are in a nitrogen atmosphere. The total concentration of transition metal ions for both the aqueous solution A (tank 1) and B (tank 2) is 0.075 and 1.425 mol L^−1^, respectively, and the molar ratios of Mn^2+^:Co^2+^:Ni^2+^ for solution A and B are 1:1:1 and 0:0.07:0.93, respectively. Furthermore, solution A is equal to solution B in volume. At the start of the co-precipitation procedure, Ni-rich solution (tank 2) was firstly fed at a constant rate of 100 mL h^−1^ into the reactor containing a certain amount of distilled water and NH_3_·H_2_O at 50 °C. Simultaneously, the solution A (tank 1) was pumped into tank 2 at a constant rate of 50 mL h^−1^. At the same time, the mixture solution containing 1.66 mol L^−1^ NaOH and adequate quantity of NH_3_·H_2_O was added to the reactor at a reasonable rate for adjusting pH of the reaction solution to 11.5~11.8, which is suitable for formation of Ni_1−x−y_Co_x_Mn_y_(OH)_2_ precipitate. With the continuous addition of solution A to solution B, the molar ratio of Mn^2+^:Co^2+^:Ni^2+^ of solution B changes gradiently from 0:0.07:0.93 to 1:1:1; thus the concentration distribution of Ni, Co and Mn of the target precursor Ni_1−x−y_Co_x_Mn_y_(OH)_2_ precipitate will be gradient. The final concentration-gradient precipitate Ni_1−x−y_Co_x_Mn_y_(OH)_2_ consists of a Ni_0.93_Co_0.07_(OH)_2_ core, a transitional zone containing a series of Ni_1−x−y_Co_x_Mn_y_(OH)_2_ (0.07 < x < 1/3, 0 < y < 1/3) precipitate and a Ni_1/3_Co_1/3_Mn_1/3_(OH)_2_ shell. After complete addition of solutions A and B, the precipitate was aged for 15 h. Subsequently, the suspension was filtered and washed three times to get the dark green precipitate. Finally, the dark green precipitate was dried for 24 h at 110 °C in a vacuum oven. The molar ratio of total transition metal ions for solution A and solution B was 5:95 (0.075:1.425), so the chemical formula of the concentration-gradient precipitate can be simply expressed as Ni_0.9_Co_0.083_Mn_0.017_(OH)_2_ (CG-NCMOH).

The concentration-gradient LiNi_0.9_Co_0.083_Mn_0.017_O_2_ (CG-LNCM) was prepared by calcination of the mixture of LiOH·H_2_O and the dried concentration-gradient precursor Ni_0.9_Co_0.083_Mn_0.017_(OH)_2_ with a molar ratio of 1.04:1 under an oxygen atmosphere in a tube furnace in which the mixed reactants were heated with an adequate rate of 5 °C per minute to 480 °C and maintained for 5 h, and then subsequently heated to 750 °C and held for 13.5 h, and finally cooled naturally to room temperature to get concentration-gradient LiNi_0.9_Co_0.083_Mn_0.017_O_2_. For comparison, the control concentration-constant LiNi_0.9_Co_0.083_Mn_0.017_O_2_ (CC-LNCM) was prepared by the same calcination of the stochiometric mixture of concentration-constant precursor Ni_0.9_Co_0.083_Mn_0.017_(OH)_2_ (CC-NCMOH) and LiOH·H_2_O with a molar ratio of 1:1.04 under oxygen atmosphere, and the concentration-constant precursor Ni_0.9_Co_0.083_Mn_0.017_(OH)_2_ was prepared by the same co-precipitation method without tank 1, and the molar ratio of Ni^2+^, Co^2+^ and Mn^2+^ is fixed to 0.9:0.083:0.017.

### 3.2. Material Characterizations

The phases of the concentration-gradient and concentration-constant samples were investigated by X-ray diffraction (XRD, PANalytical, X’Pert3 powder) using Cu k_α_ radiation in the 2θ range of 10–80°. The morphologies of the prepared materials were observed by scanning with an electron microscope (SEM, HITACHI, SU5000) and a high-resolution transmission electron microscope (HRTEM, JEOL, JEM-2100F). X-ray photoelectron spectroscopy (XPS, Ulvac-Phi, PHI 5000 VersaProbe III) with monochromatic Al k_α_ radiation was applied to determine the oxidation state of the elements in the prepared materials.

### 3.3. Electrochemical Properties Characterizations

The 2016-type coin cell was used to evaluate the electrochemical performance of concentration-gradient and concentration-constant LiNi_0.9_Co_0.083_Mn_0.017_O_2_ electrodes by electrochemical tests. The coin cell consists of a lithium foil-counter electrode, a working electrode, a Cellguard 2500 film separator and an electrolyte of 1 mol/L LiPF_6_ in the mixed solvent of ethylene carbonate (EC), ethyl methyl carbonate (EMC) and diethyl carbonate (DEC) with a volume ratio of 4:2:4. The dried working electrodes are composed of 80 wt.% of electroactive material CG-LNCM or CC-LNCM, 10 wt.% of acetylene black and 10 wt.% of polyvinylidene fluoride, and the loading of electroactive materials is about 1.8 mg cm^−2^. The galvanostatic charge/discharge tests were carried out in the voltage range of 3.0 to 4.3 V (vs. Li^+^/Li) at 25 °C. The cyclic voltammetry (CV) was conducted on a CHI660E electrochemical workstation at 0.1 mV s^−1^ in the potential range of 3.0 to 4.3 V. To compare the diffusion coefficients of Li^+^, *D_Li_*, of CG-LNCM and CC-LNCM, the galvanostatic intermittent titration technique (GITT) was conducted on the electrodes cycled at 0.1C for three times. The GITT measurement was performed at a pulse current of 20 mA g^−1^ for 10 min, followed by a relaxation of 30 min.

## 4. Conclusions

The full concentration-gradient LiNi_0.9_Co_0.083_Mn_0.017_O_2_, in which composition varies from the Ni-rich core LiNi_0.93_Co_0.07_O_2_ to the outmost shell LiNi_1/3_Co_1/3_Mn_1/3_O_2_, was prepared by a facile co-precipitation method combined with high-temperature calcination under an inert atmosphere. Benefiting from the special functions of a concentration-gradient microstructure for which the Ni-rich core provides a high capacity, the shell supplies the excellent structural stability of the surface, and concentration-gradient distribution of compositions alleviates the formation of microcrack. Moreover, concentration-gradient LiNi_0.9_Co_0.083_Mn_0.017_O_2_ possesses a higher capacity and a better rate capability and cyclability in comparison with the concentration-constant LiNi_0.9_Co_0.083_Mn_0.017_O_2_ prepared by the same method. The combination of the concentration-gradient design and the rapid co-precipitation synthesis may provide an effective strategy for large-scale production of Ni-based ternary cathode materials with high-performance for lithium-ion batteries.

## Data Availability

Not applicable.

## Supplementary Materials

The following supporting information can be downloaded at: https://www.mdpi.com/article/10.3390/molecules28083347/s1, Figure S1: Refine XRD patterns of (a) CC-NCMOH, (b) CG-NCMOH, (c) CC-LNCM and (d) CG-LNCM; Figure S2: The EDX graph of (a) area A and (b) area B of CC-LNCM, (c) area C and (d) area D of CG-LNCM; Figure S3: Activated charge/discharge profiles of (a) CC-LNCM and (b) CG-LNCM at various current rates; Table S1: Cell parameters of as-prepared samples; Table S2: Atomic parameters of as-prepared samples; Table S3: The fitting results of fresh electrodes and electrodes after cycled for 100 cycles at 5C.
