# Supplementary material for: Outstanding Electrochemical Performance of Ni-Rich Concentration-Gradient Cathode Material LiNi0.9Co0.083Mn0.017O2 for Lithium-Ion Batteries
Source: Molecules. 2023 Apr 10;28(8):3347. doi: 10.3390/molecules28083347 (PMC10142341; doi:10.3390/molecules28083347)
Supplement: Supplementary file 1 [file molecules-28-03347-s001.zip › molecules-2329443-supplementary.pdf]

Supplementary Materials

Outstanding Electrochemical Performance of Ni-Rich Concentration-Gradient Cathode Material $LiNi_{0.9}Co_{0.083}Mn_{0.017}O_2$ for Lithium-Ion Batteries

Hechen Li [1], Yiwen Guo [1], Yuanhua Chen [2,3,*], Nengshuang Gao [1], Ruicong Sun [1], Yachun Lu [1] and Quanqi Chen [1,3,*]

1 Guangxi Key Laboratory of Electrochemical and Magneto-Chemical Functional Materials, College of Chemistry and Bioengineering, Guilin University of Technology, Guilin 541004, China
2 College of Mechanical and Vehicle Engineering, Hunan University, Changsha 410082, China
3 School of Automobile Engineering, Guilin University of Aerospace Technology, Guilin 541004, China
* Correspondence: cyh@guat.edu.cn (Y.C.); quanqi.chen@glut.edu.cn (Q.C.)

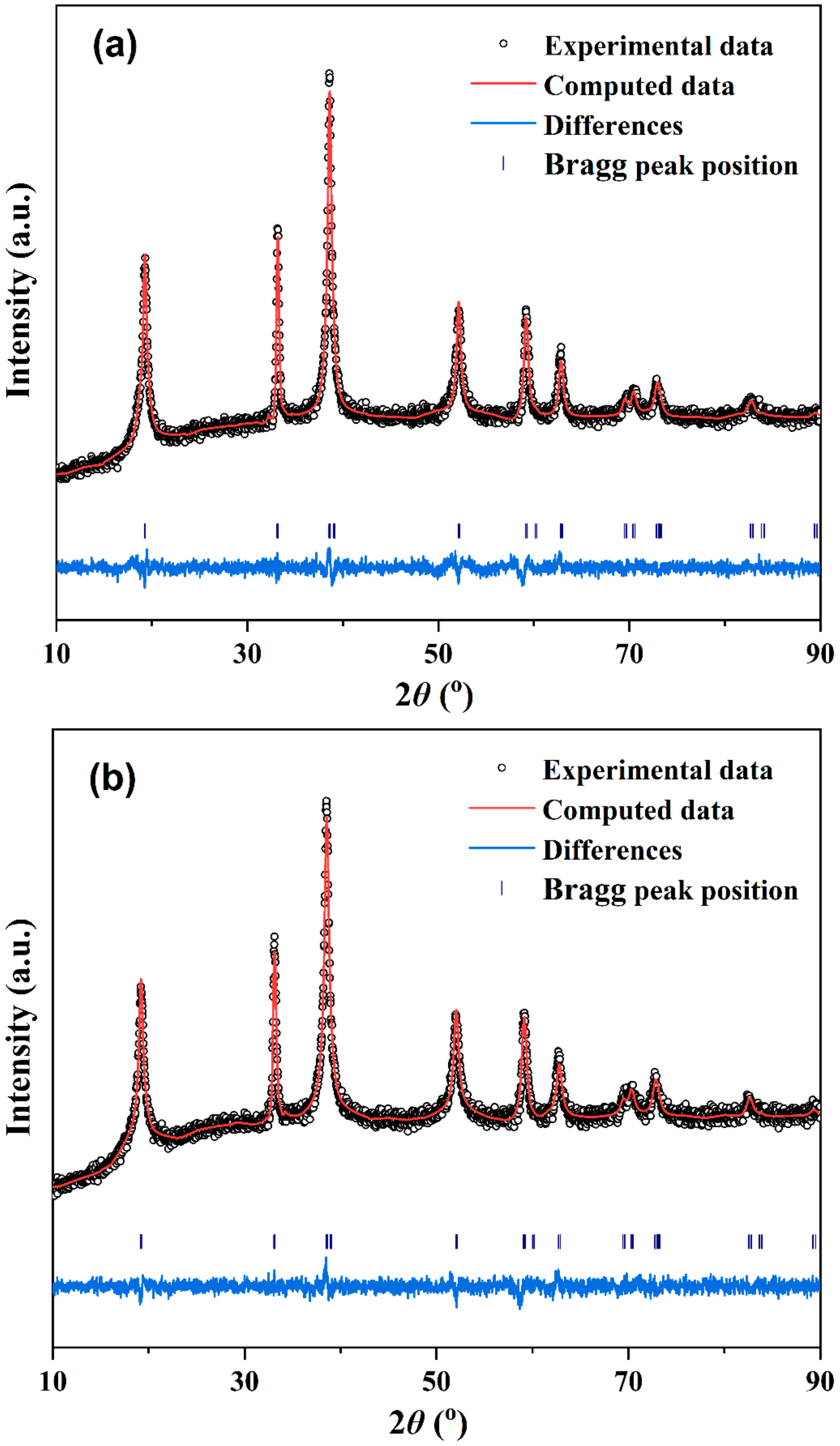
(a)
Experimental data
Computed data
Differences
Bragg peak position
Intensity (a.u.)
10
30
50
70
90
2θ (°)
(b)
Experimental data
Computed data
Differences
Bragg peak position
Intensity (a.u.)
10
30
50
70
90
2θ (°)

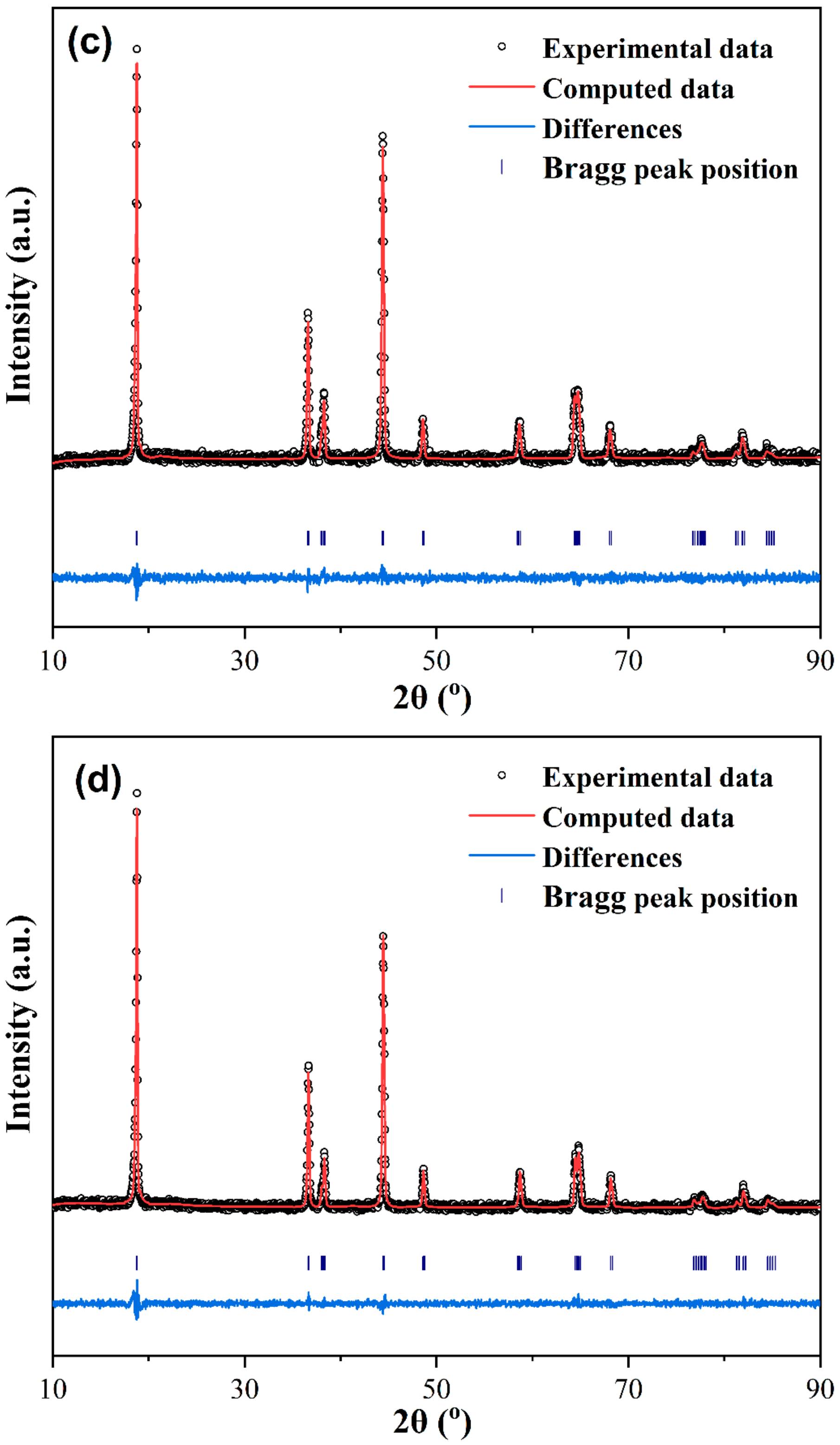

Figure S1. Refine XRD patterns of **(a)** CC-NCMOH, **(b)** CG-NCMOH, **(c)** CC-LNCM and **(d)** CG-LNCM.

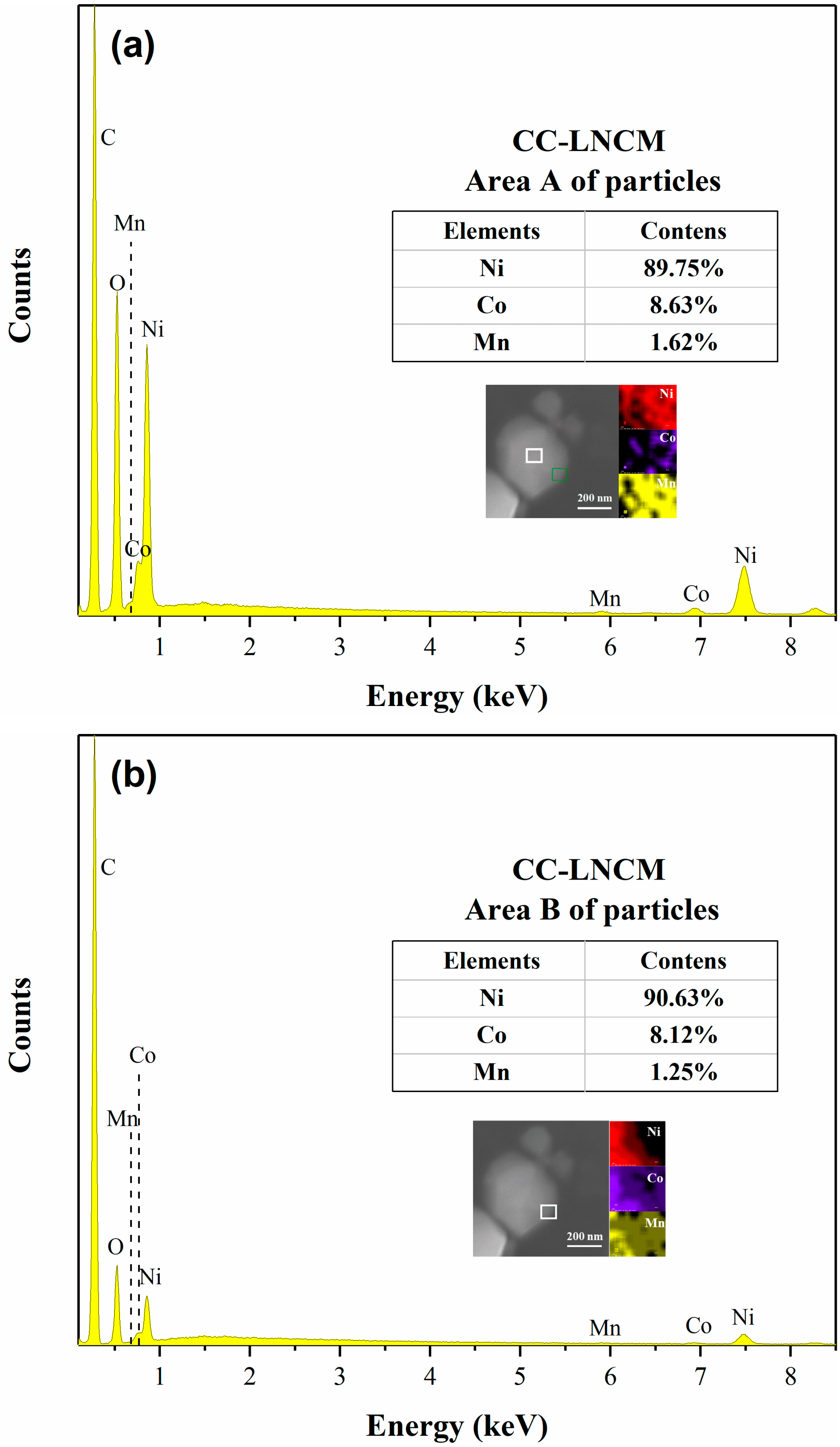
(a)
CC-LNCM
Area A of particles
Elements | Contens
Ni | 89.75%
Co | 8.63%
Mn | 1.62%
200 nm
Counts
Energy (keV)
(b)
CC-LNCM
Area B of particles
Elements | Contens
Ni | 90.63%
Co | 8.12%
Mn | 1.25%
200 nm
Counts
Energy (keV)

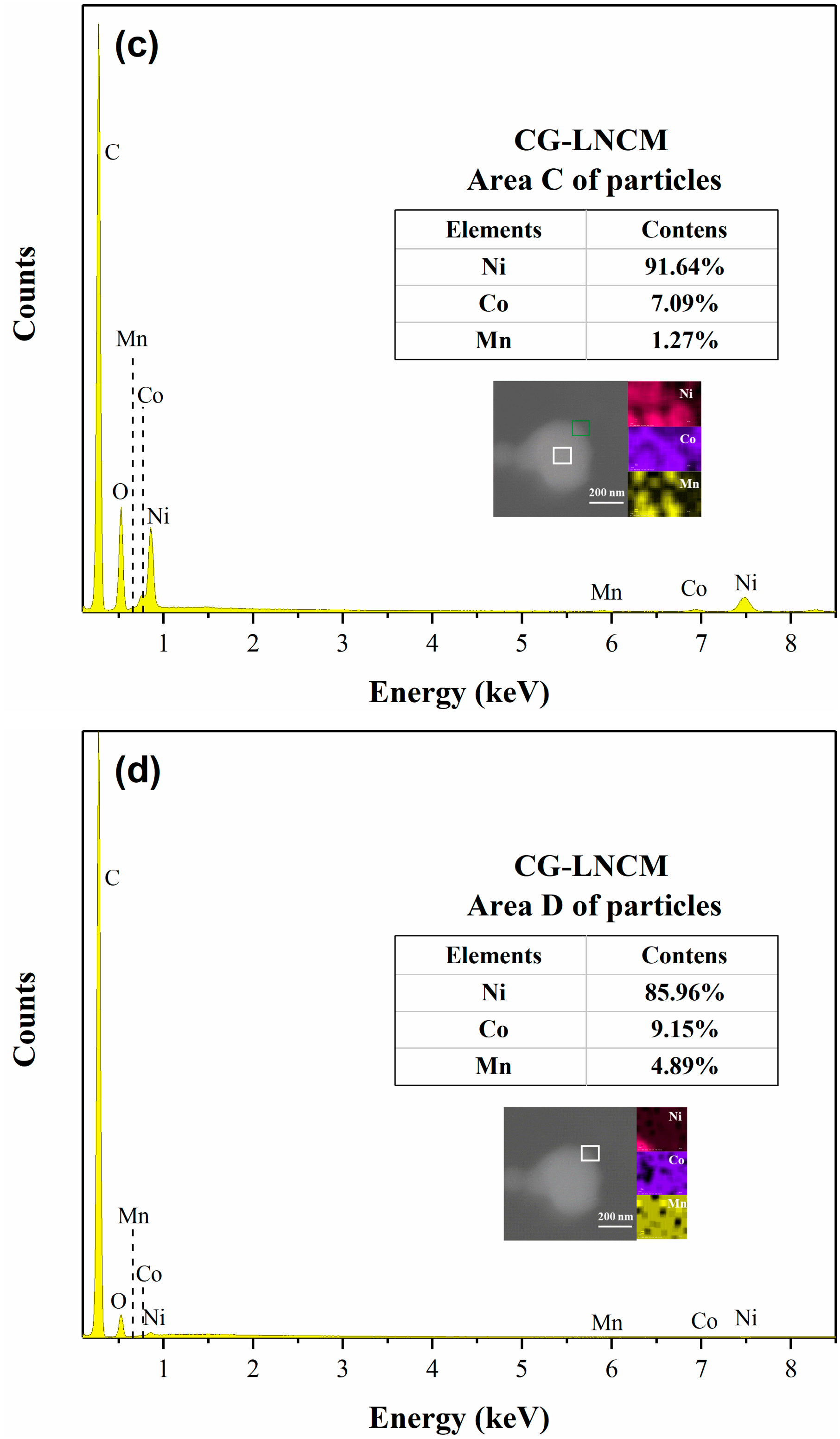

Figure S2. The EDX graph of **(a)** area A and **(b)** area B of CC-LNCM, **(c)** area C and **(d)** area D of CG-LNCM.

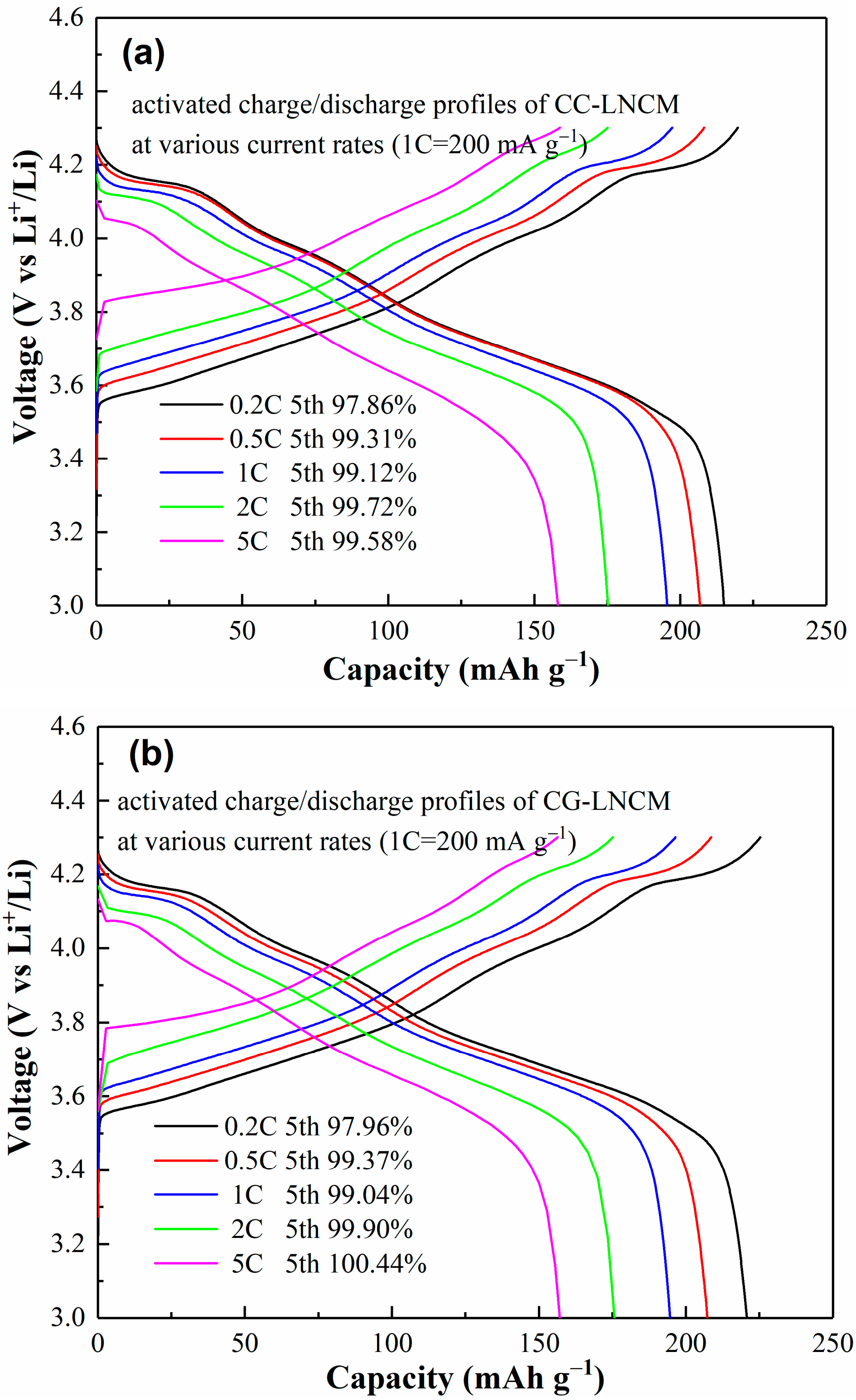

Figure S3. Activated charge/discharge profiles of **(a)** CC-LNCM and **(b)** CG-LNCM at various current rates.

Table S1 Cell parameters of as-prepared samples

sample	*a*/Å	*b*/Å	*c*/Å	*c*/*a*	$I_{(003)}/I_{(104)}$
CC-NCMOH	3.12171	3.12171	4.61164	/	
CG-NCMOH	3.12407	3.12407	4.61924	/	
CC-LNCM	2.8738	2.8738	14.1914	4.94	1.220
CG-LNCM	2.8775	2.8775	14.2059	4.94	1.395

Table S2 Atomic parameters of as-prepared samples

sample	atom	Wyckoff site	x	y	z	occupation
	Ni1	1a	0	0	0	0.900
	Co1	1a	0	0	0	0.083
CC-NCMOH	Mn1	1a	0	0	0	0.017
	O1	2d	0.33333	0.66667	0.23281	1.000
	H1	2d	0.33333	0.66667	0.43211	1.000
	Ni1	1a	0	0	0	0.900
	Co1	1a	0	0	0	0.083
CG-NCMOH	Mn1	1a	0	0	0	0.017
	O1	2d	0.33333	0.66667	0.23417	1.000
	H1	2d	0.33333	0.66667	0.43201	1.000
	Li1	3b	0	0	0.5	0.939
	Ni2	3b	0	0	0.5	0.061
	Co1	3a	0	0	0	0.083
CC-LNCM	Mn1	3a	0	0	0	0.017
	Ni1	3a	0	0	0	0.839
	Li2	3a	0	0	0	0.061
	O1	6c	0	0	0.25610	1.000
	Li1	3b	0	0	0.5	0.947
	Ni2	3b	0	0	0.5	0.053
	Co1	3a	0	0	0	0.083
CG-LNCM	Mn1	3a	0	0	0	0.017
	Ni1	3a	0	0	0	0.847
	Li2	3a	0	0	0	0.053
	O1	6c	0	0	0.25820	1.000

Table S3 The fitting results of fresh electrodes and electrodes after cycled for 100 cycles at 5C.

electrode	R_f (Ω)	R_{ct} (Ω)
fresh CC-LNCM electrode	——	62.9
fresh CG-LNCM electrode	——	48.0
cycled CC-LNCM electrode	109.6	253.0
cycled CG-LNCM electrode	44.2	186.9
